# Nasal-type NK/T-cell lymphoma with concurrent myocardial and central nervous system involvement: A rare case report

**DOI:** 10.1097/MD.0000000000045817

**Published:** 2025-11-14

**Authors:** Dan Huang, Chunyan Wu, Fucen Liu, Ziyi Zhao, Yi Tao, Zhao Kang, Chuandong He

**Affiliations:** a Department of Nuclear Medicine, Mianyang Central Hospital, School of Medicine, University of Electronic Science and Technology, Mianyang, China; b Department of Oncology, Fulin Hospital, Mianyang, China.

**Keywords:** central nervous system involvement, myocardium, NK/T-cell lymphoma, positron emission tomography-computed tomography

## Abstract

**Rationale:**

Extranodal NK/T-cell lymphoma primarily occurs in the nasopharynx, oropharynx and upper gastrointestinal tract. Involvement of the myocardium and central nervous system (CNS) is rare and poses significant diagnostic challenges. However, due to its rarity, the clinical course and optimal management of such advanced cases remain poorly characterized. This case report aims to address this knowledge gap by presenting a patient with nasal NK/T-cell lymphoma who developed both myocardial and CNS involvement, highlighting the diagnostic pitfalls and the critical importance of early suspicion.

**Patient concerns:**

A 32-year-old male with nasal subtype NK/T-cell lymphoma who was clinically diagnosed with myocardial invasion through abnormal findings on ^18^F-fluorodeoxyglucose (^18^F-FDG) positron emission tomography-computed tomography (PET-CT). During the later stages of chemotherapy, the patient confirmed CNS infiltration through laboratory diagnosis.

**Diagnoses:**

Nasal-type NK/T-cell lymphoma with concurrent myocardial and CNS involvement.

**Interventions:**

DDGP was selected as the first-line chemotherapy regimen, and famciclovir was administered for anti-EBV therapy.

**Outcomes:**

In the early phase of chemotherapy, the patient demonstrated significant clinical improvement. However, during the later course of chemotherapy, the patient was diagnosed with CNS involvement by lymphoma and succumbed shortly thereafter.

**Lessons:**

This case underscores that in patients with stage III or IV NK/T-cell lymphoma, lymphoma invasion should be the primary suspicion when cardiac or CNS abnormalities arise. Our case emphasizes the imperative for heightened clinical vigilance, prompt imaging and tailored treatment strategies are critical to potentially improve survival in such advanced cases.

## 1. Introduction

NK/T-cell lymphoma is an aggressive malignancy that demonstrates a strong predilection for Asian and South American populations.^[1]^ It is predominantly extranodal, with common sites of involvement including nasopharynx, oropharynx, and upper gastrointestinal tract. In other nonnasal cases, typical presentation sites include the skin, urethra, testes and salivary glands.^[2]^ Cardiac involvement by lymphoma occurs more frequently in of B-cell non-Hodgkin lymphoma, particularly diffuse large B-cell lymphoma, and typically manifests as a right heart mass. Cardiac involvement by NK/T-cell lymphoma is exceedingly rare and is associated with high mortality due to heart failure.^[3]^ Diagnosing lymphoma-related myocardial infiltration remains challenging, as patients are frequently asymptomatic and pathological confirmation is difficult, most confirmed cases are identified postmortem.^[4]^ Similarly, central nervous system (CNS) involvement in NK/T-cell lymphoma is uncommon, reported in 0% to 11% of patients.^[5]^ As with cardiac infiltration, obtaining pathological evidence for CNS involvement is difficult, and its management differs from that of systemic disease.^[6]^

Here we present a rare case of nasal-type NK/T-cell lymphoma with sequential involvement of both the myocardium and CNS. Lymphoma-related myocardial infiltration was initially detected by ^18^F-fluorodeoxyglucose (^18^F-FDG) positron emission tomography-computed tomography (PET-CT), which revealed characteristic metabolic abnormalities. Subsequent CNS involvement was confirmed via cerebrospinal fluid (CSF) analysis during chemotherapy. The patient developed shock several days after diagnosis of CNS involvement and succumbed despite aggressive resuscitation. Given the exceptional rarity and diagnostic challenges associated with such presentations, this case underscores the critical importance of early imaging and biochemical profiling to detect occult infiltrates. We thus provide a comprehensive analysis of this case to enhance clinical vigilance regarding potential cardiac and CNS infiltration in NK/T-cell lymphoma.

## 2. Case report

### 2.1. Patient information

A 32-year-old male was admitted with complaints of unexplained left nasal obstruction accompanied by ipsilateral facial swelling and low-grade fever. The patient denied any pain, dizziness, facial motor dysfunction, or olfactory/gustatory disturbances. He worked as a clerical officer with no known occupational exposure to industrial toxins, particulate matter, or ionizing radiation, and had no history of malignancy. He reported a 10-year smoking history averaging 3 cigarettes per day (0.15 pack-year) and a comparable duration of alcohol consumption averaging approximately 50 g of ethanol daily.

### 2.2. Clinical findings and diagnostic assessment

**Figure 1.**
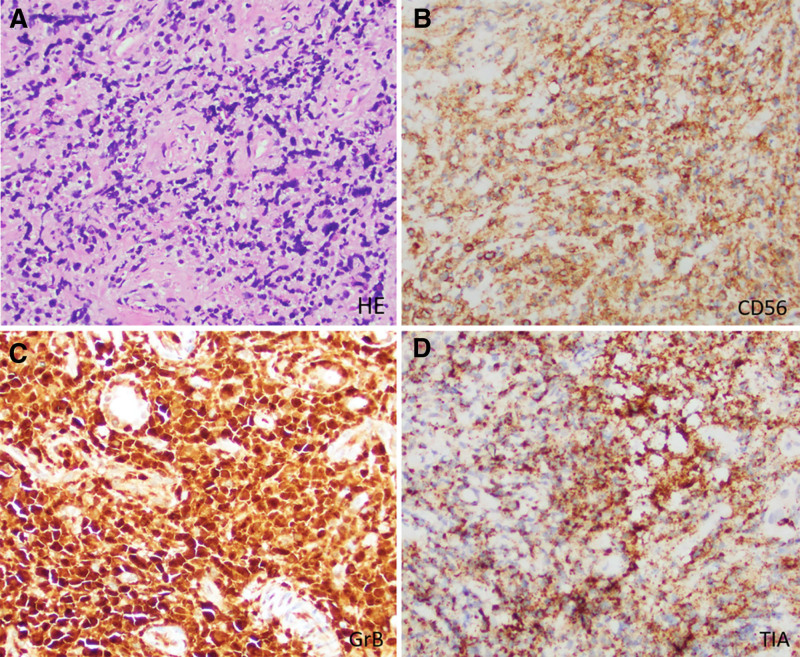
Representative images of pathological examination. (A) Hematoxylin and eosin staining image (×200): diffuse distribution of atypical lymphocytes. (B–D) The immunohistochemical staining images (×200): positive for CD56, GrB, TIA.

CT revealed a soft tissue mass in the left maxillary sinus. The patient underwent local palliative resection, and the pathological diagnosis confirmed nasal-type NK/T-cell lymphoma. Postoperative pathological examination showed a diffuse infiltrate of atypical lymphocytes (Fig. 1A). Immunohistochemically, tumor cells were positive for CD56, GrB, TIA (Fig. 1B–D). EBV-DNA was positive (33,000 copies/mL) in peripheral blood.

**Figure 2.**
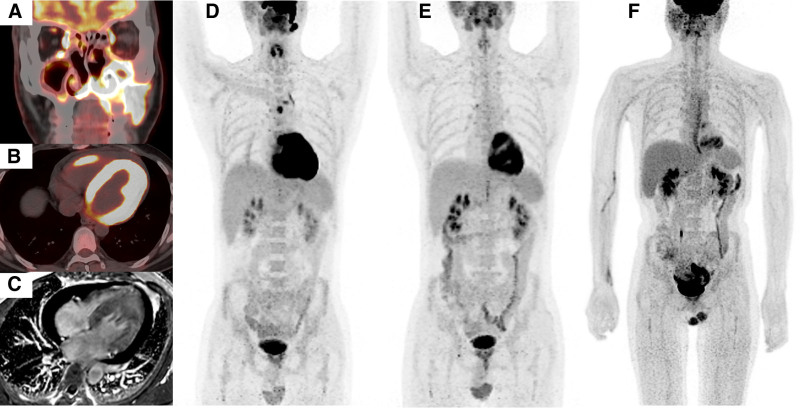
Representative images of cardiac and primary lesion. (A and B) PET/CT fusion images: lesions with increased glucose metabolism in the nasal and facial regions (SUVmax: 32.1), increased local glucose metabolism in the right ventricular wall, left ventricular wall and interventricular septum (SUVmax:37.8). (C) CMR imaging: Mild thickening of the left ventricle and interventricular septum, with delayed enhancement of the left ventricular wall. (D) PET MIP: increased local glucose metabolism in heart and Mediastinal lymph nodes have been showed. (E) PET MIP after 2 cycles of chemotherapy: The range of nasal-facial lesions was reduced with a decrease in glucose metabolism (SUVmax: 6.2), and a significant decrease in glucose metabolism in mediastinal lymph nodes and the ventricular wall (SUVmax: 12.7). (F) PET MIP after 5 cycles of chemotherapy MIP: the nasal-facial and mediastinal lesions have completely relieved, and myocardial glucose metabolism has decreased compared to the previous time. CMR = cardiac magnetic resonance, MIP = maximum intensity projection, PET = positron emission tomography.

Pretreatment ^18^F-FDG PET-CT scan (Fig. 2A, B, D) demonstrated multiple hypermetabolic lesions involving the nasal-facial region, right ventricular wall, left ventricular wall and interventricular septum. Enlarged mediastinal lymph nodes were also observed. The distribution and metabolic features of these lesions were highly suggestive lymphoma-related infiltration. Although cardiac morphology appeared normal, the presence of myocardial hypermetabolism on ^18^F-FDG PET-CT posed a diagnostic challenge. While these findings may indicate rare cardiac involvement by NK/T-cell lymphoma, myocarditis could not be ruled out. Further diagnostic evidence was required to exclude inflammatory cardiac disease.

Echocardiography showed mild left atrium enlargement, pericardium effusion, impaired left ventricular diastolic function, and preserved systolic function. These findings lacked specificity for distinguishing between cardiac lymphoma and myocarditis. Cardiac magnetic resonance imaging (Fig. 2C) revealed mild thickening of the left ventricle wall. However, due to the patient’s tachycardia, image quality was suboptimal, thus, cardiac magnetic resonance findings were only suggestive of myocardial involvement, primarily in the left ventricle and interventricular septum. Electrocardiogram showed Sinus rhythm at 90 beats per minute, with ST-segment elevation (0.05–0.20 mV) in leads aVL, II, III, aVF, and V4 to V6 and ST depression in V1 to V2. Myocardial enzyme profiling showed: creatine kinase-MB (22.86 μG/L), hypersensitive troponin T (414 ng/L), consistent with myocardial injury. None of examinations provided conclusive evidence for a definitive differential diagnosis.

Due to patient’s refusal to undergo endomyocardial biopsy, pathological examination of myocardial involvement was not obtained. Given the absence of clinical features suggestive of myocarditis, the overall presentation was highly indicative of lymphoma-related myocardial infiltration. Follow-up PET-CT after chemotherapy was recommended for reassessment of cardiac involvement.

### 2.3. Therapeutic intervention

DDGP (cisplatin 30 mg on days 1–4, dexamethasone 25 mg on days 1–5, gemcitabine 1.4 g on day 1 and day 8, pegylated asparaginase 3750 U on day 1) was selected as first-line chemotherapy, and fanciclovir was used for anti-EBV. The timeline is summarized in Table 1.

**Table 1:** Timeline.

One months prior to admission	no obvious cause of left nasal congestion, accompanied by ipsilateral facial swelling and low-grade fever. accompanied by left facial swelling and low fever. CT indicated a soft tissue mass in the left maxillary sinus, proceeded local palliative resection and was diagnosed with nasal NK/T-cell lymphoma.
Day 1	PET/CT demonstrated lesions with increased glucose metabolism in the nasal and facial regions, increased local glucose metabolism in ventricular wall and interventricular septum.
Day 3-Day 7	Echocardiography showed mild enlargement of the left atrium. CMR imaging revealed mild thickening of the left ventricle and interventricular septum, with delayed enhancement of the left ventricular wall. Abnormal electrocardiogram and myocardial markers indicate damage to myocardial cells. EBV-DNA was positive in peripheral blood.
Day 8	Began to use the antitumor therapy regimen of DDGP. Fanciclovir tablets for anti-EBV treatment.
Day 28	The PET-CT findings confirmed the efficacy of anti-lymphoma therapy. Electrocardiogram abnormalities showed significant improvement. Anti-EBV treatment demonstrated clinical effectiveness. The final diagnosis was lymphoma with myocardial infiltration, allowing for precise lymphoma staging.
Day 75	During the fifth cycle of chemotherapy, the patient experienced unclear speech and right deviation of the tongue extension.
Day 77	Head MRI examination suggested abnormal signals in the right hippocampus, left insula, bilateral cerebellar hemispheres, and vermis.
Day 90	After 14 days of symptomatic treatment, the patient’s clinical symptoms showed no improvement. MRI findings demonstrated progression of intracranial lesions.
Day 97	PET-MRI showed the nasal and mediastinal lesions have completely relieved, intracranial abnormal signal with increased glucose metabolism, considered as autoimmune encephalitis. Corticosteroids and immunoglobulins are utilized as pharmaceutical interventions.
Day 118	NK cells and EB virus were detected in cerebrospinal fluid, confirming a diagnosis of CNS involvement by NK/T-cell lymphoma. Given the patient’s physical condition (KPS = 20) at this time, which makes them unable to tolerate antitumor therapy, palliative and symptomatic care remains the primary treatment approach.
Day 125	The patient’s consciousness impairment deepened, with a decrease in blood pressure, heart rate, and finger pulse oxygen saturation. Later, vital signs disappeared, and clinical death was announced.

CMR = cardiac magnetic resonance, MRI = magnetic resonance imaging, PET-CT = positron emission tomography-computed tomography.

### 2.4. Follow-up

Post 2 cycles of chemotherapy, ^18^F-FDG PET-CT (Fig. 2E) revealed regression of nasofacial lesions and significantly reduced metabolic activity in mediastinal lymph nodes and myocardium. Cardiac biomarkers declined and EBV-DNA became undetectable. Clinical improvement confirmed myocardial involvement, leading to a stage IV (Ann Arbor) diagnosis. Two further DDGP cycles achieved partial remission.

**Figure 3.**
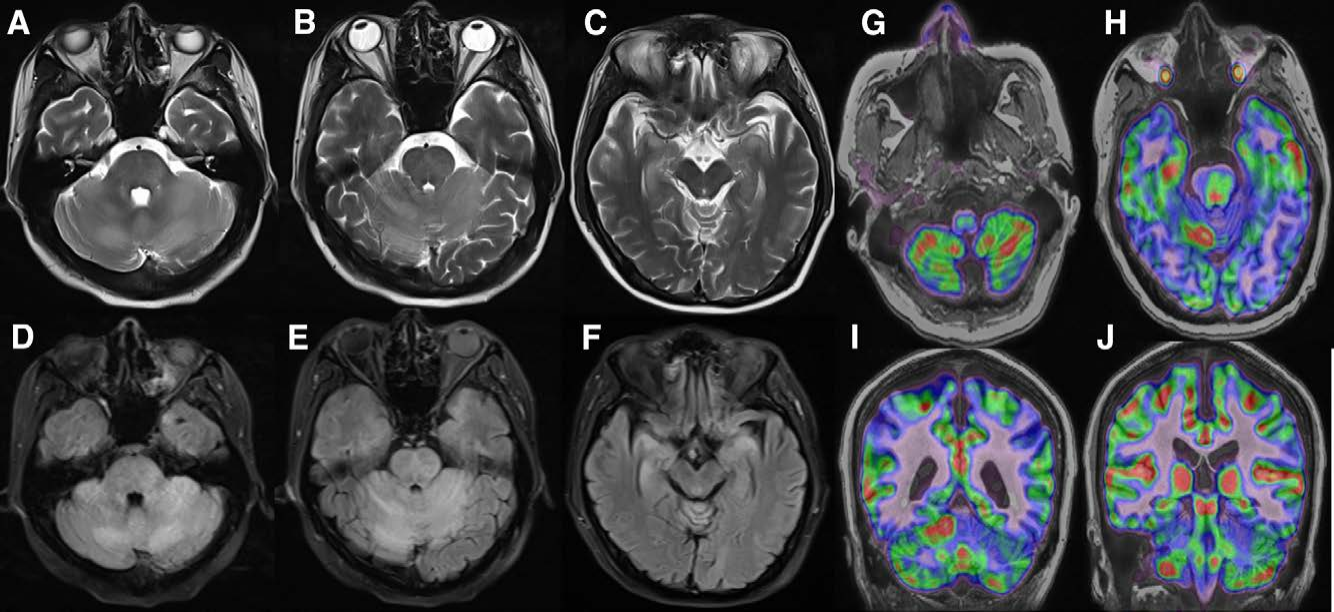
Representative images of brain lesion. (A–C) MRI T2WI, (D–F) MRI T2Flair: High signal in the right hippocampus, left insula, bilateral cerebellar hemispheres, and vermis, and the possibility of borderline encephalitis. (G–J) PET/CT fusion images: Intracranial abnormal signal with increased glucose metabolism, considered as autoimmune encephalitis. MRI = magnetic resonance imaging, PET = positron emission tomography, CT = computed tomography.

During the fifth cycle, neurological symptoms emerged including dysarthria and gait instability. MRI (Fig. 3A–F) showed expanding T2/FLAIR hyperintensities in multiple regions. Subsequent PET-MRI demonstrated complete metabolic resolution of original lesions (Fig. 2F) but new intracranial hypermetabolism (Fig. 3G–J). CSF flow cytometry detected abnormal NK cells (35.5%), and EBV was identified in the CSF, confirming CNS involvement by NK/T-cell lymphoma.

### 2.5. Outcomes

The patient’s physical condition at the time precluded tolerance to further chemotherapy. Supportive care included immunomodulatory agents, anti-inflammatory therapy, gastrohepatic protection, electrolyte correction, and nutritional support. One week later, the patient’s impaired consciousness progressed to a deep coma, accompanied by progressive decline in blood pressure, heart rate, and peripheral oxygen saturation. Ultimately, vital signs became undetectable, and clinical death was declared.

## 3. Discussion

This report presents an exceptionally aggressive case of nasal-type NK/T-cell lymphoma marked by sequential involvement of the cardiac and CNS – a pattern of progression that has rarely been documented in the literature. The patient’s clinical course, from the initial nasofacial manifestations to fatal multiorgan dissemination, highlights the considerable diagnostic and therapeutic challenges associated with advanced-stage NK/T-cell lymphoma.

### 3.1. Cardiac involvement

Cardiac involvement by lymphoma occurs more frequently in the right side of the heart and typically exhibits invasive, intramural, and pericardial growth patterns, which may manifest as solitary or multiple masses. In this case, however, the patient exhibited normal myocardial morphology without mediastinal masses or pericardial thickening. Such presentations are relatively uncommon and have been documented primarily in diffuse large B-cell lymphoma.^[7]^ Conventional imaging techniques, including ultrasonography and CT, often provide limited diagnostic value,^[8]^ posing diagnostic challenges. Here, ante-mortem detection of lymphoma-related myocardial infiltration was achieved using ^18^F-FDG PET-CT.^[7]^ The PET-CT showed abnormal increase in myocardial glucose metabolism, prompting clinical concern regarding cardiac involvement. However, that significant physiological variations in myocardial glucose uptake complicate the interpretation of PET-CT findings; thus, this modality alone cannot definitively confirm lymphomatous infiltration. In this patient, pathological sampling of myocardial tissue was deemed unfeasible due to the high risk and technical challenges, precluding histopathological confirmation. Nonetheless, following 2 cycles of chemotherapy, a progressive decline in cardiac biomarkers, resolution of electrocardiographic abnormalities, and reduced myocardial glucose uptake on follow-up PET imaging collectively supported the diagnosis of lymphoma-associated myocardial involvement.

### 3.2. CNS involvement

The clinical symptoms of CNS involvement of lymphoma are nonspecific, and imaging features can resemble those of autoimmune encephalitis, making differential diagnosis dependent on laboratory support. Li et al^[9]^ analyzed 414 patients with NK/T-cell lymphoma, CNS involvement was uncommon, accounting for approximately 4.59%. Patients with CNS involvement had a poor prognosis, with a median survival time of about 17 months. It is worth noting that some patients in Li study did not undergo CNS MRI, potentially leading to an underestimation of the number of patients with CNS involvement. In the present case, lymphoma involving the CNS was identified during chemotherapy, and CSF analysis confirmed CNS dissemination before death. The primary, mediastinal, and cardiac lesions all showed tumor regression after treatment, whereas the intracranial lesion was the only site that demonstrated progression. Therefore, we hypothesize that the progression of the intracranial lesion may have been the primary cause of the patient’s death. Without intracranial tumor involvement, the patient’s survival would likely have been significantly prolonged. This case thus underscores the importance of multimodal surveillance in advanced-stage NK/T-cell lymphoma, suggesting that occult extracutaneous involvement may be more common than clinically apparent. Moreover, the progression of CNS disease during chemotherapy – despite improvement in systemic and cardiac lesions – emphasizes the role of the CNS as a potential sanctuary site and highlights the need for prophylactic or early intervention strategies in high-risk cases.

### 3.3. Therapeutic challenges

Since the incidence rate of NK/T-cell lymphoma involving the CNS is relatively low, large-scale clinical studies addressing its risk factors and standard treatment schemes remain lacking. In this case, the patient was managed with DDGP chemotherapy – a regimen associated with favorable systemic response rates in advanced NK/T-cell lymphoma (overall response rate approximately 95%, complete remission approximately 71%, 1 year progression-free survival approximately 86%, 1 year overall survival approximately 90%).^[10]^ In this case, the DDGP regimen which used to treat the patient with poor efficacy. This outcome invites reflection on alternative strategies – such as CNS-penetrant regimens or prophylactic intrathecal therapy – for patients with high-risk features (e.g., multiorgan involvement, high EBV load).^[6]^

### 3.4. Efficacy evaluation

EBV infection is highly correlated with NK/T-cell lymphoma. According to the diagnostic criteria established by the World Health Organization, in addition to standard histopathological features, NK/T-cell lymphoma must be EBV-positive.^[11]^ PET-CT and plasma EBV-DNA assessment serve as effective dynamic prediction methods during the first 2–3 cycles of treatment.^[12]^ In this report, the observed rapid clearance of EBV-DNA and initial metabolic response are consistent with previous reports underscoring the utility of plasma EBV-DNA and PET-CT as dynamic prognostic indicators.

### 3.5. Limitations

The principal limitation of this case report remains the lack of histological confirmation of cardiac involvement by lymphoma, which was due to the patient’s refusal based on concerns about potential risks. Moreover, as a single case, generalizability is inherently limited. Biopsy is the gold standard for definitively confirming cardiac involvement by lymphoma. However, based on the patient’s clinical manifestations and imaging evidence, cardiac lymphoma involvement was clinically diagnosed. Even without undergoing cardiac biopsy, the patient still received first-line treatment for NK/T-cell lymphoma, and subsequent regression of the lesions following treatment supported the accuracy of our diagnosis.

## 4. Conclusion

This case exemplifies a highly aggressive form of NK/T-cell lymphoma with sequential myocardial and CNS dissemination. It underscores the importance of careful monitoring for neurologic or cardiac symptoms in advanced NK/T-cell lymphoma.

## Author contributions

**Data curation:** Yi Tao.

**Investigation:** Dan Huang, Ziyi Zhao, Yi Tao, Zhao Kang.

**Methodology:** Fucen Liu, Chuandong He.

**Resources:** Zhao Kang.

**Writing – original draft:** Dan Huang, Fucen Liu.

**Writing – review & editing:** Chunyan Wu, Chuandong He.
